# Non-Recurrent Laryngeal Nerve: A Rare Anatomical Entity in a Patient Undergoing Hemithyroidectomy

**DOI:** 10.7759/cureus.29935

**Published:** 2022-10-05

**Authors:** Manisha Dash, Prasad Deshmukh, Sagar S Gaurkar, Chandra Veer Singh, Ajinkya Sandbhor

**Affiliations:** 1 Otolaryngology-Head and Neck Surgery, Jawaharlal Nehru Medical College, Datta Meghe Institute of Medical Sciences, Wardha, IND; 2 Otolaryngology-Head and Neck Surgery and Surgical Oncology, Jawaharlal Nehru Medical College, Datta Meghe Institute of Medical Sciences, Wardha, IND

**Keywords:** case report, colloid goitre, dysphagia lusoria, dyspnoea, aphonia, hemi-thyroidectomy, non-recurrent laryngeal nerve

## Abstract

A non-recurrent laryngeal nerve (NRLN) is a common anatomical modification with an occurrence rate ranging from 0.5% to 0.7% in surgical procedures related to thyroid pathology [1]. In this condition cervical vagus nerve reaches the larynx directly, increasing the likelihood of vocal cord palsy. Non-RLN injury can be reduced by anticipating it and identifying it early. This case report describes how a non-recurrent inferior laryngeal nerve was discovered intraoperatively during systemic dissection, averting intra-operative nerve injury. A 40-year-old female reported to the department of Otorhinolaryngology and Head and Neck Surgery Outpatient Clinic for a nodular tumor in her neck that has been increasing for the previous five years. The colloid multi-nodular thyroid of the right lobe was confirmed by a fine needle aspiration cytology (FNAC). The patient was lined up for a surgical procedure requiring resection of the right lobe of the thyroid. A non-recurrent right inferior laryngeal nerve was discovered during surgery. The operation and recovery went smoothly, and there was no change in his voice in subsequent follow-ups. For those who are related to this professional line, this presentation provides a summary of what a non-recurrent laryngeal nerve looks like during surgery. This is critical for anyone undergoing diagnostic and surgical procedures which demand to be invasive in the region involving the neck and upper thorax, as it lowers the risk of iatrogenic nerve injury. A solitary trauma of this nerve can induce irreversible hoarseness, whereas a multilateral lesion might result in aphonia and potentially deadly dysphonia.

## Introduction

The inferior laryngeal nerve (ILN) is designated as recurrent because it loops upward post branching from the vagus nerve, on the right advances beneath the subclavian, and the left underneath the ligamentum arteriosum [1]. Excluding the cricothyroid muscle, the inferior laryngeal nerve innervates all intrinsic muscles of the larynx. It innervates the laryngeal mucosa lower than the level of the vocal cords from a sensory standpoint [2]. As a result of this nerve injury, the ipsilateral vocal cord may be paralyzed, leading to persistent hoarseness. This brings to light the anatomical positioning of the fixed vocal cords, which might cause blockade of the glottis if the lesion is bilateral; thereafter, life-threatening dyspnea and aphonia follow [3]. The inferior laryngeal nerves' relatively long course pushes it to a risk of iatrogenic injury in a variety of upper thoracic and cervical procedures [1]. Surgical operations in the cervical region, such as thyroidectomies, are among the most prevalent. Indefinite injury to the recurrent laryngeal nerve (RLN) has been documented in research to date ranging from 0.25 to 2.6 percent of cases in this last situation, with rates exceeding 8% in the case of reoperation [4]. Exploration and viewing of the ILN during procedures of this sort have been shown to dramatically lower the risk of damage to this nerve [5]. To do so, you must have a thorough understanding of both the conventional and aberrant arrangements of the ILN [6]. We present a case of an incidental intraoperative finding of the non-recurrent laryngeal nerve (NRLN) in a patient undergoing hemithyroidectomy without any other syndicated anomalies with an intention to throw light on this variant of ILN and help surgeons to be more vigilant during dissection in thyroid surgeries.

## Case presentation

**Figure 1:**
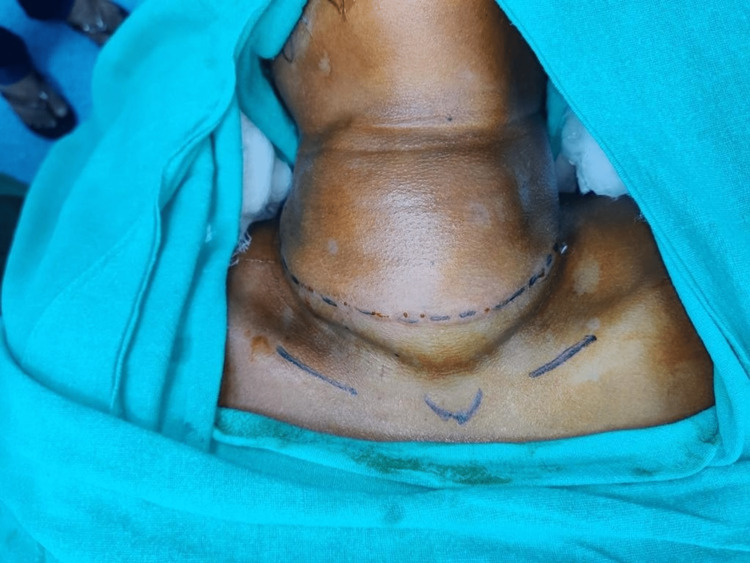
Midline neck swelling

A 40-year-old female approached the ENT and Head and Neck Surgery department with a mass in her neck that had been growing for five years, initially as small as a pea and gradually increased to a size of 10*5cm. It was a midline swelling presenting over anterior aspect of neck and moving with deglutition. It was extending from cricoid cartilage to suprasternal notch inferiorly, and lateral border of clavicular heard of sternocleidomastoid muscle (SCM) on either side with well-defined borders (Figure 1).

Her previous medical history was unremarkable. Computed tomography (CT) revealed large heterogeneously enhancing mass arising from bilateral lobes of thyroid predominately right lobe with bilateral upper middle and lower jugulodigastric and posterior cervical lymphadenopathy.

**Figure 2:**
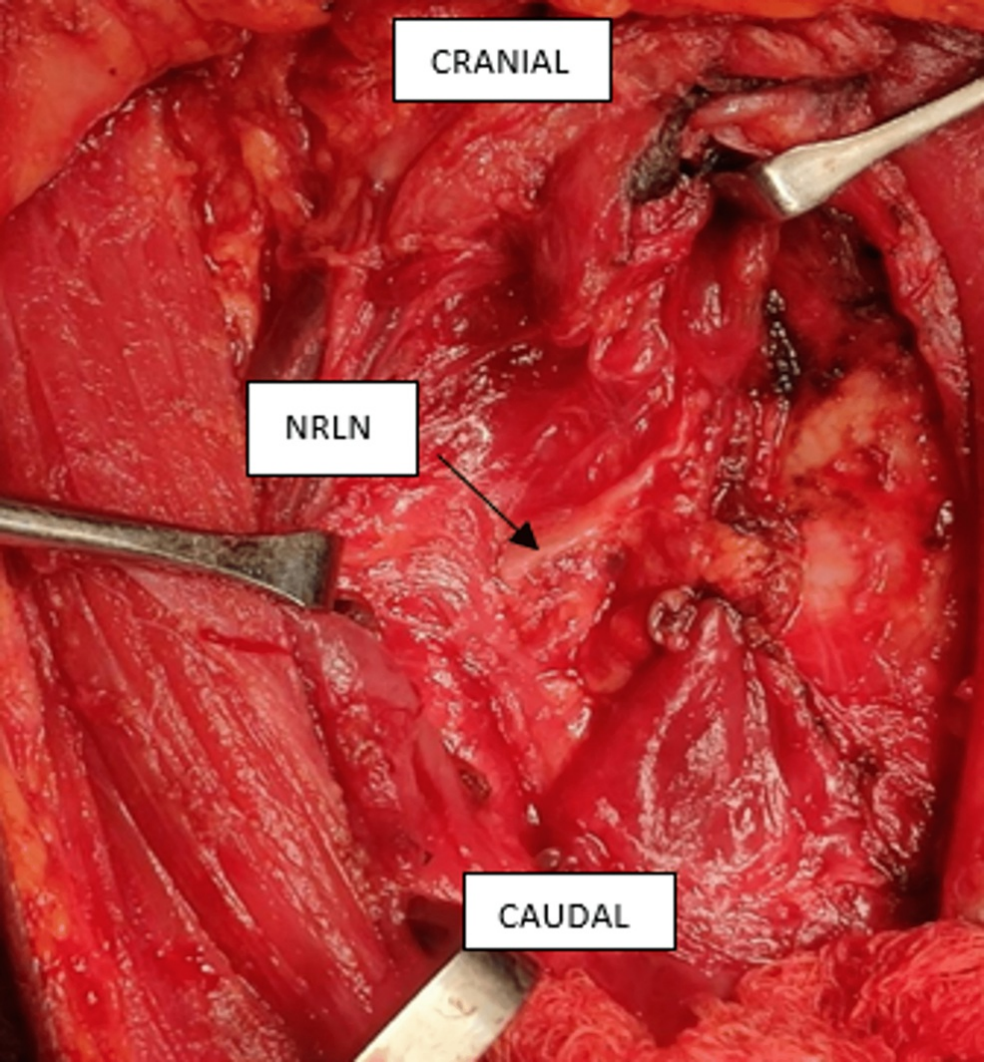
Non-recurrent laryngeal nerve shown by the black arrow is seen entering the larynx at a right angle without descending.

Right hemithyroidectomy was carried out, during which a non-recurrent right inferior laryngeal nerve was noteworthy. This nerve was seen to originate from the vagus nerve on the right side at an angle of ninety degrees and innervated the larynx 3 cm after its origin (Figure 2). Meticulous dissection was done to avoid the slightest possible trauma to the nerve. The surgery and recovery went smoothly, and our patient’s voice remained unchanged.

**Figure 3:**
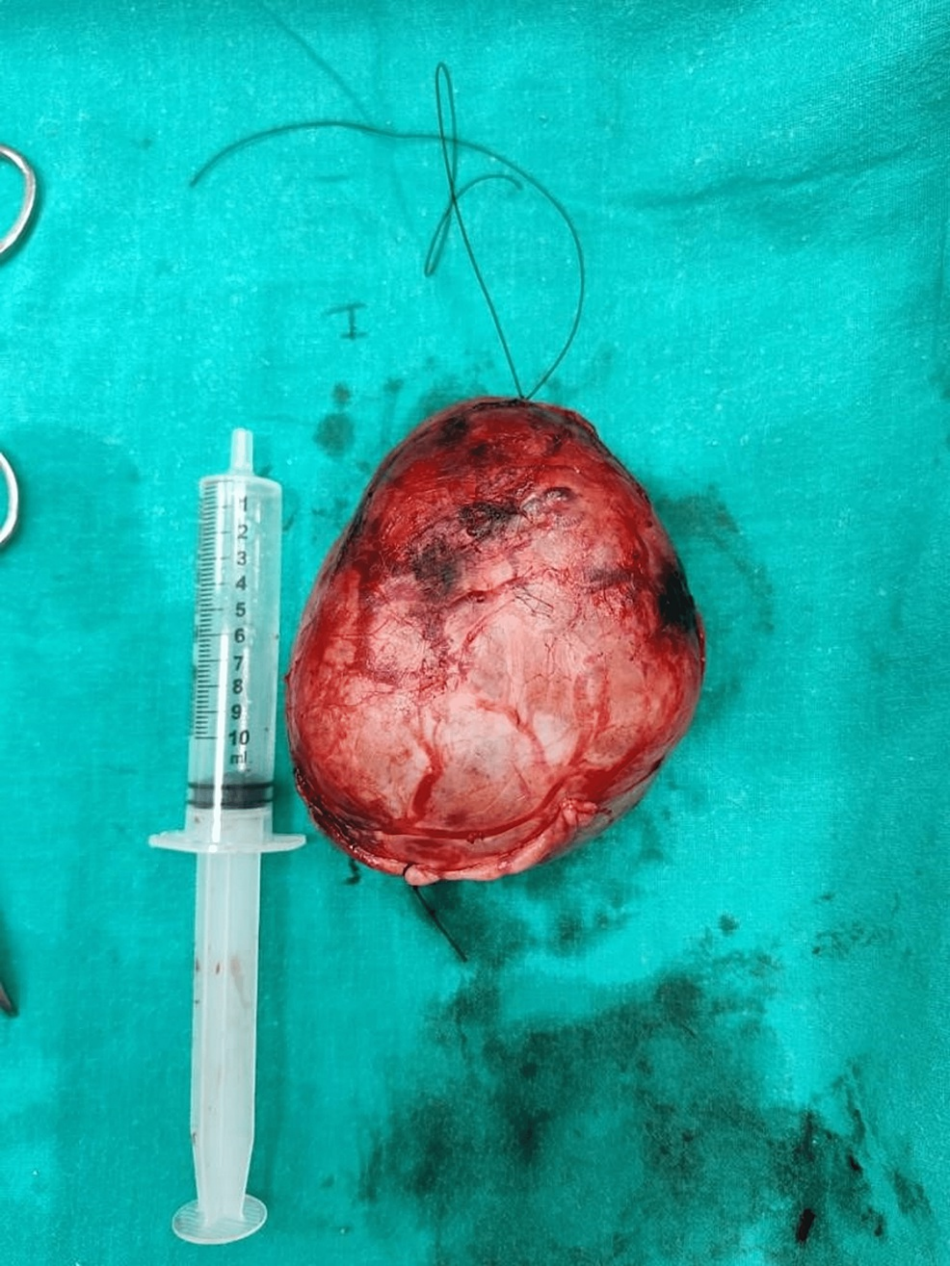
Colloid goitre removed after meticulous dissection

FNAC preoperatively revealed nodular colloid goiter, which was later confirmed with histopathological reports of the specimen postoperatively (Figure 3).

## Discussion

Non-recurrent ILN has a wide range of fluctuating incidence. The fraction of this variation is nil in certain series [3-7], whereas in others, it is measured up to 3.9% [8-10]. In one of the longest series described, ILN was observed in 6637 patients undergoing neck surgery; the count of the non-recurrent ILN was 0.54 percent (17 instances in 3098) on the right and 0.07 percent (2 cases in 2846) on the left, equal to a global frequency of 0.32 percent [11]. Despite similarity in the recurrence of this ILN variant, most authors concluded higher prevalence of this anatomical variation on the right side.

As the heart descends beneath the 6^th^ aortic arch, the 6^th^ branchial arch nerve also referred to as the RLN, climbs to the larynx. The distal portions of the 5^th^ and 6^th^ aortic arch vanish as the nerve ascends beneath the 4^th^ aortic arch on the right side, becoming a long-standing subclavian channel. Following the disappearance of the arch, the subclavian course emerges straight from the aorta (absent subclavian artery or arteria lusoria), further away from the typically situated cleared-out subclavian supply channel. The nerve occasionally travels cranially and emerges as an NRLN straight from the vagus [9]. Up to 0.5-2 percent of the general population may be afflicted by this vascular abnormality, according to research. Although it is asymptomatic in most cases, about 5% of these patients experience difficulty in swallowing, otherwise known as dysphagia lusoria, or other symptoms as a result of blood vessel tortuosity, early atherosclerosis, or, in extremely rare cases, aneurism development [10]. Numerous congenital cardiovascular issues, as well as chromosomal and other diseases, have been linked to it, which was not seen in our present case as the patient underwent a detailed clinical examination before the procedure, which shadows the probability of the presence of NRLN. In addition to having a right-sided common carotid trunk, thoracic duct, and NRLN, arteria lusoria is characterized by an aberrant root of the right vertebral artery from the aorta or the right common carotid artery. In the population, it is unusual to have both an arteria lusoria and a bicarotid trunk. Another variant of the aortic arch branching out from this trunk, which emerges from the aortic arch and bifurcates into the right and left common carotid arteries [11]. In accordance with Piersol's [12] taxonomy of aortic anomalies, which categorizes them into five categories, this example belongs to group 2, which includes the right aberrant subclavian artery, and group 5, which includes the common trunk of common carotids. This is the very first time these vascular malformations have been related to an NRLN in humans; these anatomical alterations have only ever been documented in cadavers. Frequently connected to the situs inversus or a right aortic arch, the NRLN is prevalent on the left side [9]. There are also three different types of NRLN: Type 1, which describes the nerve traveling along the superior vascular pedicle of the thyroid gland; Type 2, where the nerve travels adjacent to the superior vascular pedicle of the thyroid gland and Type 3, where the nerve travels along the superior vascular pedicle of the thyroid gland. In Type 2a, it follows the inferior thyroid artery's main trunk, and in Type 2b, it runs underneath the inferior thyroid artery's main trunk or in between its branches [13].

It was possible to functionally detect the vagus and laryngeal nerves utilizing intraoperative neurophysiological monitoring IONM [14] in a recommended investigation that involved intraoperative neuromonitoring of the vagus nerve (VN) and the RLNs.

The Nerve Integrity Monitor (NIM) was used for IONM (NIM-Response 3.0 System; Medtronic Xomed, Jacksonville, FL, USA). The stimulation intensity was set to 1 mA, and the amplitude threshold was set to 100 V on the device. After visual identification of the nerves following a precise dissection technique, they were activated immediately. The process for IONM was broken down into four steps. R2 (Post-dissection RLN Stimulation), V2 (Post-dissection VN Stimulation), RLN Dissection Technique, and Non-RLN Dissection Technique. V1 (Pre-dissection (VN) Stimulation, R2 (Post-dissection RLN Stimulation), V2 (Post-dissection VN Stimulation). Preoperative radiological analysis to rule out any anatomical diversification of any tissue dissections intraoperatively, as well as motor activity and perseverance structures determination by IONM, is a critical step in avoiding damage to vital structures and operative complications related to that damage.

Giving prominence to post-operative hoarseness of voice without association with noisy breathing in the form of stridor or aspiration symptoms which excludes the possible damage to the RLN, despite the nerve being identified and preserved during surgery, is a common complaint of the majority of patients. Available literature supports that this can be well managed with steroid administrations and vocal cord exercises as prescribed by a speech therapist, and in the rare case can take up to six months to resolve.

## Conclusions

Although NRLN is a rare occurrence, failure to recognize it can lead to iatrogenic ligation and the complications following it. An NRLN should be suspected in a patient proposed for thyroid surgery if he has a preoperative diagnosis of dysphagia lusoria or situs inversus. Even though there were no underlying anatomical abnormalities in our case, which decreases the probability of the occurrence of deviation of the course of nerve from normal, the RLN should be structurally and meticulously dissected longitudinally in the area of the tracheoesophageal groove, and in its absence, transversely in the fascial space between the carotid sheath and the larynx to preserve this vital structure and avert difficulties from occurring.
